# Digital Undergraduate Education in Dentistry: A Systematic Review

**DOI:** 10.3390/ijerph17093269

**Published:** 2020-05-07

**Authors:** Nicola U. Zitzmann, Lea Matthisson, Harald Ohla, Tim Joda

**Affiliations:** Department of Reconstructive Dentistry, University Center for Dental Medicine Basel, University of Basel, 4058 Basel, Switzerland; lea.matthisson@unibas.ch (L.M.); h.ohla@unibas.ch (H.O.); tim.joda@unibas.ch (T.J.)

**Keywords:** dental education, digital dentistry, augmented reality (AR), virtual reality (VR)

## Abstract

The aim of this systematic review was to investigate current penetration and educational quality enhancements from digitalization in the dental curriculum. Using a modified PICO strategy, the literature was searched using PubMed supplemented with a manual search to identify English-language articles published between 1994 and 2020 that reported the use of digital techniques in dental education. A total of 211 articles were identified by electronic search, of which 55 articles were selected for inclusion and supplemented with 27 additional publications retrieved by manual search, resulting in 82 studies that were included in the review. Publications were categorized into five areas of digital dental education: Web-based knowledge transfer and e-learning, digital surface mapping, dental simulator motor skills (including intraoral optical scanning), digital radiography, and surveys related to the penetration and acceptance of digital education. This review demonstrates that digitalization offers great potential to revolutionize dental education to help prepare future dentists for their daily practice. More interactive and intuitive e-learning possibilities will arise to stimulate an enjoyable and meaningful educational experience with 24/7 facilities. Augmented and virtual reality technology will likely play a dominant role in the future of dental education.

## 1. Introduction

The implementation of digital technologies in dental curricula has started globally and reached varying levels of penetration depending on local resources and demands. One of the biggest challenges in digital education is the need to continuously adapt and adjust to the developments in technology and apply these to dental practice [1]. Most dental offices in Europe are equipped with software solutions for managing patients’ records, agenda and recall reminders; recording provided services, including working time schedules; ordering materials; and managing the maintenance contracts of medical devices. These systems incorporate medical histories, digital radiographs, intraoral photographs, medicine lists, and correspondences. The systems also enable easy access to detailed odontograms showing fillings per tooth surface, restorations and carious lesions, periodontal status with visualization of the attachment level, probing pocket depth, and recession [2].

The introduction of intraoral optical scanning (IOS) allows the current anatomic situation to be digitized, enabling chairside or laboratory fabrication of restorations, to plan oral rehabilitations with a set-up [3], and/or to superimpose the situation with 3-dimensional (3D) radiography (e.g., for guided implant placement) [4]. While the penetration of these scanners in dental offices is still limited (present in an estimated 20%–25% of European dental offices) [5], laboratory scanners are presumably used by more than two-thirds of dental laboratories. The dental technician uses the 3D model files derived from IOS by the clinician or from scanned conventional casts to facilitate the fabrication of restorations. Compared to waxing, the digital design offers several advantages for quality control, such as providing data about material thickness and values of connector cross sections. While the main shortcomings of lost wax casting were erroneous castings or shrinkage cavities, with a digital workflow the laboratory benefits from improved material properties when industrially manufactured products can be used with subtractive milling or additive printing processes [6].

3D education programs have been introduced to enhance students’ spatial ability, their interactivity, critical thinking, and clinical correlations with the integration of multiple dental disciplines. Augmented reality in 3D visualization allows insights in tooth morphology, and also facilitates treatment planning with fixed or removable partial denture (RPD) programs [7]. Digital technologies also include the 3D printing of virtual teeth, which has been suggested to enhance transparency for all students due to the identical setups [8].

A recent review on the application of augmented reality (AR) and virtual reality (VR) in dental medicine demonstrated that the use of AR/VR technologies for educational motor skill training and clinical testing of maxillofacial surgical protocols is increasing [9]. It was concluded that these digital technologies are valuable in dental undergraduate and postgraduate education, offering interactive learning concepts with 24/7 access and objective evaluation. A recent scoping review analyzed the application of VR in pre-clinical dental education and identified four educational thematic areas (simulation hardware, realism of simulation, scoring systems, and validation), highlighting the need for a better evidence base for the utility of VR in dental education [10]. In communicating with dental professionals, medical doctors, dental technicians, and insurance providers, dental students have to be prepared to manage digitized data, ensure patient safety, and understand the benefits and limitations of conventional and digital processes.

Overall, digitalization seems to have had a major impact on dental education, addressing various aspects, such as e-learning and Web-based knowledge transfer, but also related to diagnostics using 3D imaging and digital radiography, and practically oriented trainings in terms of dental simulator motor skills including IOS with 3D printing, prototyping, and digital surface mapping. Digital applications can provide additional opportunities to evaluate and improve education, implementing evidence-based surveys related to the penetration and acceptance of digital education.

The aim of this systematic review was: (i) to investigate the current level of implementation of digital technology in dental education; and (ii) to outline the educational quality enhancements that result from digitalization in main focus areas within the dental curriculum.

## 2. Materials and Methods 

This systematic review was conducted in accordance with the guidelines of Preferred Reporting Items of Systematic Reviews and Meta-Analyses (PRISMA) [11]. A systematic electronic search of PubMed was performed, limited to English-language articles published between 1 January 1994 and 15 April 2020. A modified PICO search was defined for Population/TOPIC, Intervention/METHOD, and Outcome/INTEREST; whereas Comparison was omitted. The search syntax used was: ((students[MeSH]) AND (education, dental[MeSH] OR teaching[MeSH] AND digital)) AND (dentistry[MeSH] OR dental medicine). In addition, the bibliographies of all full texts selected from the electronic search were manually searched, and an extensive search of articles published in the *Journal of Dental Education* and the *European Journal of Dental Education* was conducted.

This systematic review focused on randomized controlled trials, cohort studies, case–control studies, observational trials, and descriptive studies that investigated the application of digital technologies in dental education. Reports without an underlying study design and studies not involving dental students were not included. Furthermore, the vast body of literature about the transition from glass to digital slide microscopy was also excluded. Four reviewers (N.U.Z., T.J., L.M., H.O.) independently screened the titles, abstracts, and the full texts of the identified articles to select those for inclusion in the review. Disagreements were resolved by discussion. Duplicates or preliminary reports that were followed by original publications were excluded. 

## 3. Results

**Figure 1:**
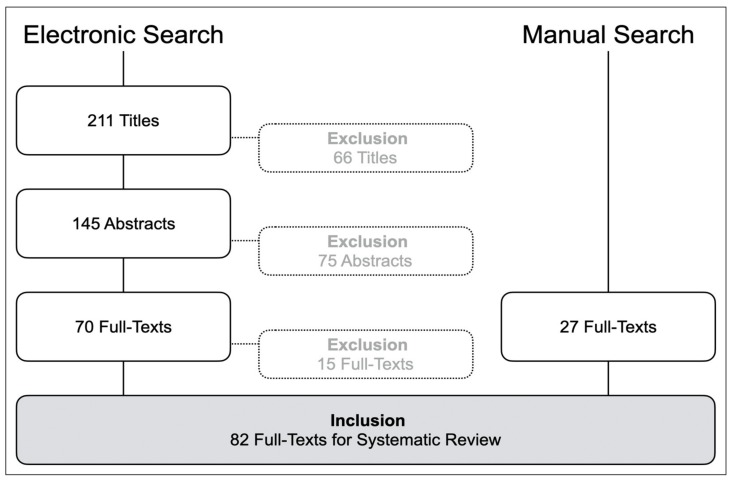
Systematic search strategy.

A total of 211 titles were identified by the electronic search (Figure 1). After screening of the titles, abstracts, and full-text articles, 55 publications were included that reported a digital application in dental education. The manual search retrieved 27 additional publications, resulting in the inclusion of 82 studies (Annex S1 and Annex S2).

The publications were categorized into six areas of digital dental education:

- Web-based knowledge transfer/e-learning (22 studies);
- Digital surface mapping (20 studies);
- Dental simulator motor skills including IOS (23 studies);
- 3D printing and prototyping (2 studies);
- Digital radiography (5 studies); and
- Surveys related to the penetration and acceptance of digital education (10 studies).

### 3.1. Web-Based Knowledge Transfer/e-Learning 

Fifteen studies reported the use of Web-based learning tools in the dental curriculum, comprising orthodontics [12,13], tooth anatomy [14,15,16], oral pathogens and immunology [17], dental radiology [18,19], oral surgery [20] or implant dentistry [21], prosthetic dentistry [22], caries detection [23,24], in growth and development [25], and the general use of Web-based learning tools [26] (Table 1). Three additional studies reported on the use of video illustrations of clinical procedures with behavior management in pediatric dentistry [27], intraoral suturing [28], or tooth preparation [29]. Practicing history-taking and decision-making in periodontology with a Web-based database application, where students used free text communication on the screen to interact with patient data, improved their capability and empathy during the first patient contact [30]. One other study described the introduction of portable digital assistants for undergraduate students in a primary dental care clinic to access a virtual learning environment; these tools proved to be a convenient and versatile method for accessing online education [31]. Mobile devices were found to support learning by offering the opportunity to personalize digital learning materials by making comments, underlining, annotating images, and making drawings [32]. The availability of free 3D viewer software favored the planning of RPD designs on 3D virtual model situations [33]. Online access to digital tools without time restrictions was identified as a major benefit in dental education, and Web-based instructional modules facilitated students’ individual learning approach and accommodated varying learning paces. While an initial effort was required to prepare online educational material, faculty time was reduced in the long term.

### 3.2. Digital Surface Mapping

Visual inspection of students’ work is known to have shortcomings in inter- and intra-examiner reliability, whereas standardized digital surface mapping of abutment tooth preparations facilitates objective evaluation and feedback (Table 2) [34,35,36,37,38,39,40,41,42,43,44,45,46]. In the preclinical training of dental students, the use of software that can match the student’s scanned preparation with an ideal tooth preparation has been proven to be a helpful tool in the evaluation of preparation form, taper, and substance removal. High intra-rater agreement was also found for the repeated digital grading of wax-ups in the undergraduate curriculum [47], and students’ initial self-assessment was overrated compared to the digital grading [48]. Limitations of digital assessments have been found for intracoronal cavity preparations, due to the restricted analysis of cavity depth [49,50]. With specified software skills, successful application was documented for class II mesio-occlusal-distal (MOD) cavity assessments, class III composite preparations, and mesio-occlusal (MO) onlay preparations [51,52,53]. These studies of digital surface mapping clearly demonstrate the tremendous development of this technology since 2006, which now enables a thorough and consistent analysis of several preparation parameters, with freely available open-source comparison tools. 

### 3.3. Dental Simulator Motor Skills Including Intraoral Optical Scanning 

A high level of interest and acceptance was documented among undergraduate students for simulator training in cavity preparations [54,55,56], or in surgical interventions such as apicoectomies (Table 3) [57]. A trend toward improved technical skills and ergonomics was documented when simulator training with real-time feedback was added to traditional instructions [58,59,60]. Training with a VR-based simulator improved students’ preparation of class I occlusal cavities [61], and of abutments for porcelain-fused-to-metal crowns [62]. In evaluating the manual dexterity of students, professionals, and non-professionals, the simulator scoring algorithm showed a high reliability to differentiate between non-professionals and dental students or dentists [63]. Instruction time from faculty for teaching cavity and crown preparations was significantly reduced when virtual reality computer-assisted simulation systems were used compared to contemporary non-computer-assisted simulation systems [64]. Preparation performance on VR units with continuous evaluations and advice from clinical instructors led to better preparation quality than real-time feedback from the virtual dental unit. Self-paced learning and the immediate software feedback were beneficial with the VR unit, and it was perceived as adjunct, but not replacing faculty instructions [65]. Students requested software improvements with more realistic force feedback during interaction with different tissues in the virtual oral environment including the maxilla, mandible, gum, tongue, cheek, enamel, dentine, pulp, cementum, etc. [66]. Recent advancements of simulators enabled variations in force feedback accounting for varying hardness of the virtual material, cut speed gain, and push force [67].

Improved student performance in crown digitization and framework design was observed when CAD/CAM (Computer-Aided Design/ Computer-aided manufacturing) courses were introduced in dental education [68]. While students enjoyed designing a full crown using CAD as compared to traditional waxing, limits of the technology in representing anatomic contours and excursive occlusion were identified [69]. Viewing their scanned crown preparations magnified on the screen improved students’ understanding of the finishing line [70]. The application of IOS in the simulation training showed that even inexperienced dental students were capable of acquiring the skills needed to use digital tools, and students preferred IOS over conventional impressions [71,72]. Furthermore, students’ work time was shorter with IOS than with conventional impression [72,73], although more teaching time was required for digital scanning than for conventional impression techniques [74]. Applying digital complete denture treatment (AvaDent; AvaDent Digital Dental Solutions, Scottsdale, AZ, USA) in the student clinics resulted in restorations with superior gradings that were preferred by both students and patients [75]. Using an intraoral camera increased patients’ consent for crown treatment, and was positively perceived by students and patients, while faculty members were neutral [76].

**Table 3:** Dental simulator motor skills incl. IOS (*n* = 23).

Study (Year)	Study Design	Theory / Practice	Participants	Materials and Methods	Results
Quinn et al. 2003 [65]	RCT	P	20	Compared students’ performance in preparing class I amalgam cavity on a VR-based training unit; test group had virtual real-time feedback and software evaluation, control group had clinical instructor available during preparation. Anonymous scoring by 2 faculties, criteria: outline form, retention form, smoothness, cavity depth and cavity margin angulation. Questionnaire feed-back in test group.	Similar results for retention and wall angulation, while outline form, smoothness and cavity depth scored better in control. Test group assessed software as superior for immediate feed-back, self-paced learning, consistency of evaluation, encouraging independent work and more thorough assessment, while conventional training was superior for increasing confidence in cavity preparation. VR-based training should be used as adjunct but not replacing conventional training methods.
Jasinevicius et al. 2004 [64]	CT	P	28	Compared students’ performance in amalgam and crown preparations on typodont teeth either with a contemporary non-computer-assisted simulation system (CS), or with a virtual reality computer-assisted simulation system (VR). Both groups were provided with presentations describing preparations, CS group received handouts, VR group had preparation criteria available on the computer. Student-faculty (S-F) interaction time was logged.	Preparation quality did not differ between CS and VR. CS required 2.8 h, VR 0.5 h S-F. CS received five times more instructional time from faculty than VR.
LeBlanc et al. 2004 [60]	RCT	P	68	Compared students’ technical skills in preclinical operative dentistry after standard traditional laboratory-based instructions (over 110 h) and additional virtual reality simulator-enhanced training (test group with 20 students) Simulator (DentSim, DenX) provided real-time feedback, training conducted during 6–10 h in 3 blocks over 8 months.	While all students improved in the 4 tests during the year, test students tended to better scores in the final exam. Virtual reality simulators can be implemented in the traditional training of future dentists.
Rees et al. 2007 [54]	CT	P	16	Evaluated simulator training (DentSim, DenX) by undergraduate students for Class I and II preparations (time, marks, number of evaluations), students spent 6 h cutting an unlimited number of Class I cavities and Class II cavities; feedback by questionnaire.	Class I preparations obtained a mean mark of 66.8, preparation time was 12.5 min, with 6.7 evaluations; Class II had a mark of 26.5, time 18 min, with 7.0 evaluations. Class II was more difficult to cut. Students appreciated easy change of teeth, working at their own pace and examine the cavity in a cross-section.
Welk et al. 2008 [55]	OT	P/T	80	Evaluated students’ performance in operative dentistry after training with computer-assisted dental simulator (DentSim, DenX), feedback by questionnaire.	Students indicated high interest in simulator training, high acceptance and response to additional elective training time in the computer assisted simulation lab. The shift in curriculum and instructional goals has to be optimized continuously.
Urbankova et al. 2010 [58]	RCT	P	75	Evaluated adjunctive computerized dental simulator (CDS; DentSim) training (8 h) in operative dentistry (Class I and II preparations): either before (*n* = 26) or after 1st exam (*n* = 13); control group (*n* = 36) with traditional preclinical dental training alone (110 h).	CDS-trained students performed better than control in the 1st and 2nd exam, no difference between pre-exam and post-exam groups. In the 3rd exam (end of the year) CDS group had higher, but not significantly different scores than control.
Pohlenz et al. 2010 [57]	CT	P	53	Evaluated VR training (Voxel-Man) for virtual apicoectomy; questionnaire about simulated force feedback, spatial 3D perception, resolution and integration of further pathologic conditions.	92.7% recommended the virtual simulation as additional modality in dental education, 81.1% reported the simulated force feedback as good or very good, 86.8% evaluated 3D spatial perception as good or very good; 100% recommended integration of further pathologies.
Gottlieb et al. 2011 [59]	CT	T	202	Evaluated VR simulation training (DentSim, Image Navigation Ltd.) in operative preparations and restorations, 60 h VR training, laboratory course was reduced to 234 h (instead of traditional 304h). 13 experienced faculties assessed 97 non-VR students (1st year, control) and 105 students with 1 semester VR experience (test); survey about students’ abilities in ergonomics, confidence level, performance, preparation, and self-assessment.	Faculty expected greater psychomotor skills and ability to prepare teeth in VR, abilities were lower than anticipated but numerically higher than in non-VR students. Faculty members perceived students’ ergonomics in the test group better than in control.
Ben-Gal et al. 2011 [56]	CT	P	33	Evaluated use of VR simulator (IDEA Dental) for dental instruction, self-practice, and student evaluation. 21 experienced dental educators, 12 randomly selected experienced dental students (5th year) performed 5 drilling tasks using the simulator, feed-back by questionnaire.	Both groups found that the simulator could provide significant benefits in teaching and self-learning of manual dental skills.
Ben-Gal et al. 2013 [63]	CT	P	106	Evaluated potential of VR training simulator (IDEA Dental) to assess manual dexterity in 63 dental students, 28 dentists, 14 non-dentists, performed virtual drilling tasks in different geometric shapes: time to completion, accuracy, number of trials to successful completion, score provided by the simulator.	Simulator scoring algorithm showed high reliability in all parameters and was able to differentiate between non-professionals and dental students or non-professionals and dentists.
Lee & Gallucci 2013 [73]	CT	P	30	Compared digital (IOS) to conventional impression for single implant restorations, evaluated efficiency, difficulty and students’ preference.	Mean total treatment time, preparation time and working time were significantly longer for conventional than for IOS; conventional impressions were assessed as more difficult than IOS; 60% preferred IOS, 7% conventional, 33% either techniques
Kikuchi et al. 2013 [62]	RCT	P	43	Compared VR simulator (DentSim) training with or without instructor feedback for preparation of porcelain fused to metal (PFM) crown preparation. 43 students (5th year). randomly divided into: 1. VR group with instructor’s feedback (DSF; *n* = 15); 2. VR without instructor’s feedback (DS; *n* = 15); 3. neither VR simulator training nor faculty feedback (NDS; *n* = 13); preparation time and scores of 4 crown preparations (1week for 4 weeks).	DSF and DS had significantly higher total scores than NDS. Similar results in DSF and DS, but shortened preparation time with instructors’ feed-back (DSF) at early stages.
Douglas et al. 2014 [69]	CT	P	50	Compared students’ performance in traditional waxing vs. computer-aided crown designing (IOS with CEREC 3D, Sirona Dental Systems), faculty grading of occlusal contacts and anatomic form, feed-back by questionnaire.	Similar gradings for wax design (79.1) and crown design (78.3); more occlusal contacts with CAD; students enjoyed designing a full contour crown using CAD and required less time with CAD. Students recognized limits of CAD technology in representing anatomic contours and excursive occlusion compared to conventional wax techniques.
Wang et al. 2015 [66]	CT	P	20	Compared VR simulator (iDental with Phanotm Omni, SensAble Tech. Inc.) in novice group (graduate students with less than 3 years clinical practice experience) and resident group (with 3–0 years clinical practice); assessment of caries removal, pulp chamber opening, time and amount of removed healthy/unhealthy tissue; feed-back by a questionnaire.	No differences in time and amount of tissue removal between groups; residents spend slightly more time than students; both groups suggested improvements in spatial registration precision, more realistic model with material properties and force feedback of different tissues, improvement of the depth of the virtual space.
Schwindling et al. 2015 [68]	CT	P	56	Evaluated a CAD/CAM hands-on course (test) compared to video-supported lecture only (control); written exam about cast digitizing and zirconia crown designing.	Test group performed significantly better than controls (16.8/20 vs. 12.5/20 correct answers); interest of students in CAD/CAM was higher after hands-on course.
Kattadiyil et al. 2015 [75]	CCS	P	15	Compared clinical treatment outcomes, patient satisfaction, and dental student preferences for digital (AvaDent, two appointments) and conventional (five appointments) complete dentures (CD) in 15 patients, 15 dental students fabricated two sets of CDs for each patient. Faculty and patient ratings, patient and student preferences, perceptions, treatment time was analyzed.	Digital process was equally effective and more time-efficient than conventional; faculty scored digital better than conventional dentures; patients and students preferred digital dentures.
Zitzmann et al. 2017 [72]	RCT	P	50	Investigated performance (time recording) and perception (questionnaire feedback) of IOS and conventional implant impression after video teaching.	Students rated conventional impressions as more difficult (VAS 46) than IOS (VAS 70), with greater patient-friendliness of IOS (VAS 83) compared to conventional impressions (VAS 36); 76% preferred digital, 88% felt most effective with IOS; total work time of all steps was significantly shorter with 301 sec. for IOS and 723 sec. for conventional impressions.
Wegner et al. 2017 [70]	OT	P	108	Evaluated students’ perception (questionnaire feedback) of IOS (Lava Cos Training, 3M Espe), scanning of 3 typodont tooth preparations.	63.9% positive opinion, 60.2% considered scanning process as manageable, 55.6% profited from magnified view of their preparation to understand chamfer finish lines.
Marti et al. 2017 [74]	RCT	P	25	Analyzed time to instruct IOS (DS; LAVA C.O.S. digital impression system) and conventional impression technique (CI; polyvinyl siloxane) with video lecture, investigator led demonstration, and independent impression exercise: time recording and questionnaire about familiarity and student’s expectations.	Teaching DS required significantly more time than CI for video lecture (16 vs. 10 min), demonstration time (9 vs 5 min) and impression time (18 vs. 9 min). Initially students were more familiar with CI (3.96) than DS (1.96) technique. After instructions and practice, CI technique proved significantly easier than expected. Manageability of DS was not influenced by the instruction and practice experience. 96% expressed an expectation that DS will become their predominant impression technique.
de Boer et al. 2019 [67]	RCT	P	126	Investigated skill transfer between various levels of force feedback (FFB) using Simodont dental trainer (Moog) for cross-figure preparations as manual dexterity exercise. Assessment of students’ satisfaction by questionnaire.	Longer practice time was correlated with test performance: students passing at different FFB levels had mean of 300h, those passing in one FFB level had 271 h, failing students had 224 h. Skill transfer from one level of FFB to another was feasible with sufficient training.
Schott et al. 2019 [71]	OT	P	31	Evaluated dental students’ perception of IOS compared to conventional alginate impression; survey after basic training and self-practicing.	77% (24) students were overall “very” or “rather satisfied" with the handling of IOS; 58% preferred IOS from the dentist’s perspective, no significant difference from the patient’s perspective but reduced comfort related to the impression tray.
Murbay et al. 2020 [61]	RCT	P	32	Incorporated VR with Moog Simodont dental trainer in preclinical training; students performed an occlusal preparation on typodont teeth and had previous exposure to VR (group 1) or no VR exposure (group 2); assessment was conducted (satisfactory / unsatisfactory) by manual approach or digital (Magic 19.01 64-bit).	VR use improved preparation significantly with 75% (12/16) satisfactory preparations in group 1 and 44% (7/16) in group 2. Manual and digital evaluation methods did not differ significantly.
Murrell et al. 2019 [76]	OT	P	288	Evaluated completion of posterior crown planning with or without presenting the situation to the patient by intraoral camera use; 51 students completed 198 surveys, 35 faculty members with 64 surveys, 202 patient surveys, survey was voluntary and camera use optional.	Positive perception of intraoral camera use by students and patients, while faculty was neutral; significantly higher completion rate when intraoral camera was used.

RCT = Randomized Controlled Trial; CT = Controlled Trial; CS = Cohort Study; CCS = Case-Control-Study; OT = Observational Study; DSF = VR group with instructor feedback; DS = VR group without instructor feedback; NDS = Neither VR simulator training nor faculty feedback; VAS = Visual Analog Scale; IDEA = International Dental Education Association.

### 3.4. 3D Rapid Prototyping

Two studies evaluated training models created by 3D rapid prototyping [77,78]. Such methods can supplement teaching on human teeth or even replace it, and educational needs can easily be adapted to students’ skills (Table 4).

### 3.5. Digital Radiography

Four studies dealt with diagnosing radiographic changes [79,80,81] or detecting positional errors on panoramic radiographs [82] (Table 5). Senior students showed a poor ability for approximal caries detection on both conventional and digital radiographs when compared to histo-pathologic analysis from sectioned teeth [80]. One study demonstrated that digital learning supported the development of students’ diagnostic skills [81]. Another study showed that the accuracy of radiographic caries detection was improved by a computer-assisted learning calibration program, which provided feedback illustrating the actual tooth surface condition [79]. In one study, two digital systems for endodontic tooth length measurements were compared, and students’ positive attitudes towards digital radiography were documented [83].

### 3.6. Surveys Related to the Penetration and Acceptance of Digital Education

Six surveys evaluated students’ perception and acceptance of digital technologies (Table 6) [84,85,86,87,88,89]. The more recent studies reflected that digital technologies have become established teaching tools, particularly in the field of digital radiography and microscopy, and the use of textbooks decreased; simulation training was preferred [86,87].

Four surveys analyzed the penetration of and attitudes towards digital technologies at dental schools in the UK [90], U.S. [91], North America [2], or among the faculty staff at a dental school in Malaysia [92]. According to the most recent survey, CAD/CAM technologies were taught in most dental schools in North America (93%), while other digital modalities showed less penetration [2]. 

Despite a high acceptance of digital technologies in dental education by faculty [92] and students [86], it was concluded that e-resources should not replace interactions with faculty; students wanted lectures and clinical procedures recorded [85].

## 4. Discussion

The systematic review aimed to investigate current penetration and educational quality enhancements from digitalization in the dental curriculum. Heterogeneous study types addressing various fields of digital applications were found. While a meta-analysis was not feasible, a descriptive approach for identified publications was conducted. 

Digitalization in dental education is frequently used to enhance the accessibility and exchange of documents and to facilitate the collaboration and communication among students, teachers, and administrative staff. Digitalization enables cloud-based records, evaluation, and feedback, as well as the provision of e-learning modules [23]. Students today, particularly the Millennials, expect services instantly, expect to be able to download their grades, course schedules, and other information automatically, and to be able to get assistance 24 h a day. In order to satisfy these expectations, it is necessary to promote a change of mindset of the dental faculty and provide instructors with training in e-learning and e-teaching to enable theoretical and practical knowledge transfer [85]. The coronavirus disease (Covid-19) pandemic that started in 2019 caused dental schools around the world to close, and highlighted the need for alternative channels for education (e.g., Web-based learning platforms) [93]. Scheduled webinars can provide a structure for students’ theoretical learning. Additional applications of digital features include educational videos illustrating clinical exams or therapeutic steps, interactive systems, adaptive systems that monitor students’ ability and adjust teaching accordingly, online collaborative tools, etc. The use of pictograms instead of scripts in educational videos facilitates a language-independent application in several countries.

Especially in the field of motor skills training, digital software tools can be used to evaluate the manual abilities of potential candidates for the dental curriculum, to analyze students’ preclinical preparations, to enable self-assessment, and to enhance the quality of education. The objective and exact nature of these digital evaluations helps to improve students’ visualization, provides immediate feedback, and enhances instructor evaluation and student self-evaluation and self-correction [43,94]. Students can learn to self-assess their work with self-reflection and faculty guidance in conjunction with a specially designed digital evaluation tool [48]. IOS and digital impression techniques can be included early in the dental curriculum to help familiarize students with ongoing development in the computer-assisted technologies used in oral rehabilitation [3,72].

While undergraduate students today have to be prepared for digital dentistry, they still need to acquire the knowledge of conventional treatment strategies and processes. Growing up in the digital world, they will easily adapt to digital features. Digital dentistry offers several options for an objective standardized evaluation of students’ performance, which should be used for quality enhancement. It is currently a “teaching transition time”, and new standards have to be defined for dental education in general. Open questions remain, such as: (i) in which phase of the dental curricula should digital technologies be introduced as the routine tool; (ii) which analog techniques can be omitted; and iii) which digital content should be taught in which disciplines? 

Several studies indicated that personal instruction and feedback from faculty cannot be replaced by simulator training and feedback [39,65,85]. In this context, faculty should be aware of their responsibility in teaching young dentists, who are treating individuals with individual needs requiring empathy and an informed consent for any treatment decision. Digitalization cannot replace all educational lessons or courses, and the role-model function of faculty is important when supervising students during patient treatment in the clinical courses.

It should be emphasized that there are still no uniform standards in dental education with regard to the digital tools applied. Such standards are essential to ensure uniformity in teaching, which is particularly important for an international exchange. Society as well as dentistry is currently undergoing a digital transformation. It is necessary to clarify learning contents, to what extent conventional workflows should still be taught, and what can be done digitally. While digital tools and applications in knowledge transfer are a general challenge for undergraduate education in all disciplines, the field of dentistry with its high degree of practical training units is specifically demanding. Just because training units are designed digitally does not mean that students learn on their own. Continuous training with supervision and feed-back is still the key to good dental education. In this context, digitization is certainly a great opportunity to convey the learning content with more joy and newly awakened enthusiasm.

Following the rule, “you can only teach what you are able to perform yourself”, a highly motivated faculty is needed that is willing to embrace the latest digital technologies. Besides personal motivation, the financial aspect of implementing the various digital tools and applications has to be managed at dental universities. Collaborations with industry would be helpful here. This is a classic “win–win situation”—the dental school would be equipped with the latest products and updates, and the industry would get access to the youngest target group of potential customers. In the event of such collaborations, it is vital that universities maintain their objectivity by offering a variety of products from diverse companies; otherwise, there is a risk of unduly influencing dental students and biasing them towards one particular technological option. The rapid pace of change in dental technology must also be considered. Dental technology companies are constantly introducing new products and workflows. While this provides exciting opportunities for dental research, to test and analyze those new developments, it complicates the implementation of digital workflows in dental education programs. New job descriptions are also necessary at dental schools in order to maintain the technical infrastructures required for these new technologies and to guarantee a smooth operation in clinical practice. In future, the best dental schools will be ranked according to their digital infrastructure combined with the level of innovation of the teaching faculty. 

## 5. Conclusions

Digital tools and applications are now widespread in routine dental care. Therefore, this trend towards digitization and ongoing developments must be considered in dental curricula in order to prepare future dentists for their daily work-life. There is a need to establish generally accepted digital standards of education—at least among the different dental universities within individual countries. Digitalization offers the potential to revolutionize the entire field of dental education. More interactive and intuitive e-learning possibilities will arise that motivate students and provide a stimulating, enjoyable, and meaningful educational experience with convenient access 24 h a day.

At present, digital dental education encompasses several areas of teaching interests, including Web-based knowledge transfer and specific technologies such as digital surface mapping, dental simulator motor skills including IOS, and digital radiography. Furthermore, it is assumed that AR/VR-technology will play a dominant role in the future development of dental education.

## Figures and Tables

**Table 1:** Web-based knowledge transfer / e-learning (*n* = 22).

Study (Year)	Study Design	Theory/Practice	Participants	Materials and Methods	Results
Komolpis et al. 2002 [12]	RCT	P	99	Compared effectiveness (exam scores and time spent) in clinical orthodontic diagnosis in test group (50 students with web-based digital records) and control group (49 students provided with traditional records) with study models, panoramic and cephalometric radiograph, facial and intraoral photographs.	Test and control group performed similar in the exam with no difference in test time; positive feedback about the web-based learning module, students benefit from convenient access to study material on the computer without time constrictions.
Schultze-Mosgau et al. 2004 [20]	OT	T	82	Evaluated a web-based course with a concluding online examination. Feed-back by questionnaire.	Course gradings excellent or good were given for accessibility independent of time (89%), for access independent of location (83%), for objectification of knowledge transfer (67%), and for use of videos for surgical techniques (91%).
Schittek Janda et al. 2004 [30]	RCT	P	39	Compared the effect of a web-based virtual learning environment (VLE) on students’ performance in history interview. Both groups underwent standard instruction in professional behavior, history taking, clinical decision making and treatment planning. Test group worked with the virtual periodontal patient for 1 week prior to their first patient contact; control group was first allowed to use the virtual patient after their first patient contact. Time spent, type and order of questions and professional behavior were analyzed.	Test group asked more relevant questions, spent more time on patient issues, and performed a more complete history interview than control. The use of the virtual patient and the process of writing questions in working with the virtual patient stimulated students to organize their knowledge and resulted in more confident behavior towards the patient.
Boynton et al. 2006 [27]	CS	P	108	Explored students’ behaviors management in pediatric dentistry using portable video instructions; test group: 11 students reviewing video lecture material on a portable device (iPod) supplementing conventional pediatric behavior management lecture; additional 6 students (intermediate) used audio versions or video on the computer; control group: 91 students without digital learning material; exam on student comprehension.	Test group performed significantly better on the examination (mean 9.3) than control (7.9) or intermediate group (7.8); portable format was preferred.
Reynolds et al. 2007 [31]	CS	P	12	Investigated students’ educational use of portable digital assistants (PDA) to access a Virtual Learning Environment in a primary dentalcare clinic and at home; cross over trial with 6 students with / 6 without for 12 weeks.	PDA was frequently used for online education; over 90% wanted PDA as part of their dental kit.
Kingsley et al. 2009 [17]	CS	P	78	Examined students’ ability to use web-based online technologies to find recently published online citations and to answer clinically relevant questions (oral pathogens and immunology course); technology skills analyzed: ability to locate online library resources, understand how information is organized within the library system, access online databases, interpret and evaluate research materials within the context of a specific discipline; students were provided with a review article of vaccines against caries from 2001.	100% of students had correct responses to the content-specific or technology-independent portions; 46% had correct responses to the information literacy or technology-dependent portions; as web-based technologies grow more prevalent in the digital era, information literacy and technology-dependent, applied research assignments should be integrated into graduate-level curricula.
Weaver et al. 2009 [28]	RCT	P	12	Evaluated performance in intraoral suturing after digital multimedia instruction; control group: written information; test group: plus teaching tool; suturing performed on a model situation, evaluated by 10 grading criteria.	Test group performed better than control; video addressed common mistakes made by novice students, improved long-term understanding of the basic suture principles.
Wright et al. 2009 [14]	OT	T	235	Determined whether dental students used an interactive DVD-tooth atlas as a study aid and perceived the 3D interactive tooth atlas as a value-added learning experience.	14% students downloaded the DVD voluntarily prior to adding atlas-related exam questions as incentives; after adding incentives 43% downloaded the material; financial concerns and overly sophisticated content were deemed responsible for the low acceptance.
Curnier 2010 [16]	OT	P	26	Assessed VR integration into teaching of dental anatomy, feedback by questionnaire	70% of the students were satisfied/very satisfied with IT integration in the curriculum.
Bains et al. 2010 [13]	RCT	T	90	Compared effectiveness and attitudes toward e-learning (EL, online tutorial without teacher), face-to-face learning (F2FL, led by teacher) and blended learning (BL) subdivided in BL1 (EL first then F2FL) and BL2 (F2FL first then EL) among 4th year students. Groups received cephalometric tutorial in the allocated mode, answered an MCQ (Multiple Choice Questionnaire).	F2FL and BL resulted in similar test results; EL alone was less effective. BL was the most and F2FL was the least accepted method, EL was significantly less preferred, the order B1 or 2 had no effect.
Mitov et al. 2010 [15]	CS	T	36	Testing an e-learning software (morphoDent) to prepare for an anatomy exam. 3D models with description and x-rays of permanent human teeth were available for viewing and interaction on the learning platform. Practical dental morphology exam was compared to virtual tooth anatomy exam. Evaluation of students’ perceptions in a questionnaire.	Similar exam scores in traditional and online exam. Majority felt the software helped them learning dental morphology, despite of difficulties in operating the program.
Vuchkova et al. 2012 [19]	CS	P	88	Evaluated interactive digital versus conventional radiology textbook (course radiographic anatomy), outcome was radiographic interpretation test and survey feedback.	95% perceived positive enhancement of learning and interpretation.
Smith et al. 2012 [29]	OT	P	26	Compared the use of online video-clips with traditional live demonstrations with one-to-one supervision; students exam scores before and after the video introduction were compared. Feed-back by questionnaire.	76% preferred video-clips to live demonstrations, 57% reviewed DVD at home; 57% felt one-to-one supervision more effective developing their competence in tooth preparation.
Qi et al. 2013 [21]	RCT	P	95	Comparison of active versus passive approaches in using 3D virtual scenes in dental implant cases. Students were exposed to educational materials about implant restoration on three types of webpages: traditional 2D (group 1); active-controlling 3D (group 2); passive-controlling 3D (group 3). After reviewing their webpages, students were asked to complete a posttest to assess the relative quality of information acquisition. Before study exposure, students performed a standardized test of spatial ability (mental rotations test, MRT).	Posttest scores were highest in group 3 (passive control) and lowest in group 2 (active control). Higher MRT scores were associated with better posttest performances in all three groups. Individuals with low spatial ability did not benefit from 3D interactive virtual reality, while passive control produced higher learning effects compared to active control.
Reissmann et al. 2015 [22]	OT	T	71	Creation of a blended learning model; e-learning modules covered fundamental principles, additional information, and learning tests (tests were repeated until passed and the next video sequence unlocked); modules comprised (i) tooth preparation, placement of post and core, and provisional crown; (ii) with preparation, manufacturing and insertion of a FDP (Fixed Dental Prosthesis). Students rated the course on a questionnaire, comparison to previous courses without e-learning.	Significantly higher satisfaction among students enrolled in the e-learning modules compared to the years prior to integration of the e-learning tests. Results suggest that instructor-based practical demonstrations in preclinical courses in prosthetic dentistry could be successfully replaced by e-learning applications provided that course content is structured according to specific predefined learning goals and procedures.
Luz et al. 2015 [24]	RCT	P	39	Evaluated the effect of a digital learning tool on students’ caries detection in 12 pediatric patients (3.4 per student) using ICDAS (International Caries Detection & Assessment System) (1264 dental surfaces). 2 weeks after first exam students were split into 3 training groups: Group 1: ICDAS e-learning program; group 2: plus digital learning tool; group 3: no learning strategy; students reassessed the same patients 2 weeks, and results compared.	After training group 1 and 2 had improved with significantly higher sensitivity; group 2 showed significant increase in sensitivity at the D2 and D3 thresholds as a result of the digital learning tool.
Gonzales et al. 2016 [18]	OT	T	40	Implementation social media (Twitter) in a dental radiology course and evaluated students’ use and perception by a questionnaire.	95% (38) had not used Twitter prior to the course; 53% (21) created an account during the course to view radiographic examples and stay informed; overall Twitter had a positive impact with improved accessibility to the instructor.
Jackson et al. 2018 [25]	OT	P	80	Evaluated dental students study patterns using self-directed web-based learning modules with scheduled self-study time instead of lectures; web-based module access (date and time) was recorded for four courses in the growth & development curriculum; scheduled access time was 8 am to 5 pm.	Frequency of module access (at least once) varied among the four courses (10–64%); only three students had > 20% of their total accesses taking place during designated self-study times. For all courses the proportion of module access was significantly higher 0–2 days before an exam compared to 3–7 or >7 days before final exam; no association between module access during scheduled times and course performance.
Alves et al. 2018 [23]	RCT	P	64	Evaluated the effect of a digital learning tool on students’ caries detection in 80 teeth using ICDAS; Group 1 (21 students): ICDAS e-learning program; group 2 (22 students): plus digital learning tool; group 3 (21 students): no training; reassessment of the 80 teeth 2 weeks after training.	After training group 1 and 2 had improved with significantly higher sensitivity and specificity; group 3 had increased sensitivity at the D2 thresholds; ICDAS e-learning with or without digital learning tool improved occlusal caries detection.
Botelho et al. 2019 [26]	OT	T	40	Surveyed dental students’ perception of cloud-based practice records (documenting clinical progression) compared to traditional paper record.	Cloud based records were rated significantly better in terms of usefulness, ease of use, and learning, satisfaction.
Pyörälä et al. 2019 [32]	OT	T	176	Investigated perception of mobile devices for study use among 124 medical, 52 dental students provided with iPads and followed from 1st to 5th year; feed-back by questionnaire.	Note taking was the most frequent application of the mobile device in the 1st–5th year; students personalized digital learning materials by making comments, underlining, marking images and drawings. Students retrieved their notes anytime when studying for examinations and treating patients in clinical practice.
Mahrous et al. 2019 [33]	RCT	P	77	Compared virtual 3D casts with 2D paper-based exercise in planning removable partial denture design; group 1 (*n* = 39) planned RPD in Kennedy class IV in virtual 3D and Kennedy class II in traditional 2D format, group 2 (=38) planned class IV traditional and class II virtual; survey lines and undercut positions were drawn on virtual 3D casts or given in written descriptions (2D); students planned design (with rests, clasp type, retention location, guide plane) was scored; feed-back by questionnaire.	Similar scores for 3D and 2D exercises; majority favored virtual 3D casts because of improved understanding of relevant parameters and spatial visualization. Currently, physical casts are still required to practice surveying and drawing on the cast.

RCT = Randomized Controlled Trial; CT = Controlled Trial; CS = Cohort Study; CCS = Case-Control-Study; OT = Observational Study.

**Table 2:** Digital surface mapping (*n* = 20).

Study (Year)	Study Design	Theory/Practice	Participants	Materials and Methods	Results
Esser et al. 2006 [35]	CS	P	36	Compared conventional visual examination by faculty with digital analysis (“Prep Assistant”) of students’ preparation of a central incisor for a metal-ceramic crown; preparations were scanned; before the exam preparation, students had received theoretical and practical exercises.	Digital measuring technique was superior for convergence angle, occlusal reduction and width of shoulder; low correlation between visual and digital was observed for the assessments of chamfer, path of insertion, width of bevel and basic form; calibration of evaluators benefit from digital analysis tool.
Hamil et al. 2014 [37]	OT	P	81	Evaluated dental students’ opinion about a new grading software program (E4D Compare with surface mapping technology) for their self-assessment and as faculty-grading tool in a preclinical course to evaluate crown preparations. Software was introduced (one-hour lecture and three-hour hands-on laboratory session) and applied for self-assessment during one semester; questionnaire about students’ perception.	Students preferred digital grading system over traditional hand-grading 95% reported on feedback inconsistencies among different faculty members, 72% reported on inconsistencies from the examiner; 85% agreed or strongly agreed that E4D Compare provided more consistent grading than faculty; 79% responded that the software provided more feedback, 90% found the software helping them to understand their deficiencies; 89% agreed or strongly agreed that E4D Compare grading helped them be better clinicians.
Mays et al. 2014 [49]	CT	P	25	Compared students’ visual self-assessment, students’ digital (CAD/CAM) self-assessment, faculty visual assessment, and faculty digital assessment. Students prepared mesial-occlusal amalgam cavity, used standardized grading sheets for visual self-assessment, scanned their preparation, used design tool of Cerec software for digital self-assessment.	Moderate agreement between faculty visual and digital evaluation for occlusal and proximal shape, orientation and definition; poor agreement between student visual and digital evaluation for occlusal shape, and fair for proximal shape, orientation and definition; slight to poor agreement between students visual and faculty visual evaluation, and digital assessment did not improve student/faculty agreement.
Kwon et al. 2014 [47]	OT	P	60	Compared conventional visual faculty grading of wax-ups to digital assessment in dental anatomy course; 30 faculty wax-ups, 15 student wax-ups and 15 dentoform teeth; visual grading was performed by two experienced faculty members, digital grading by one operator, both gradings were repeated after 1 week; maxillary 1st molar wax-up (from faculty) with highest scores from visual grading was used as master model for digital grading.	Modest intra-rater reliability for visual scoring with similar rating between the two trials (0.7); low inter-rater agreement between the two faculty raters; digital grading showed high intra-rater agreement for the repeated assessment (ICC 0.9); modest correlation between visual and digital grading.
Garrett et al. 2015 [48]	CCS	P	57	Evaluated E4D software (Planmeca) to assess incisor and molar wax-ups of 57 students, who used digital images for self-assessment, and compare to faculty members; based on five assessment criteria (arch alignment, proximal contacts, proximal contour and embrasures, facial contour, lingual contour) and applying 300, 400, and 500 μm level of tolerance in E4D.	Students’ self-assessment of the maxillary incisor wax-up was higher than faculty and E4D300, but lower than E4D 400 and 500. For the molar wax-up, self-assessment was not different to faculty, but higher than E4D300. E4D500 evaluations were sig. superior than other assessments.
Callan et al. 2015 [34]	CCS	P	82	Validated E4D software (Planmeca) to assess molar crown preparation of 82 students and compare to calibrated faculty members based on four criteria (occlusal reduction, proximal reduction, facial/lingual reduction, margins and draw). Agreement in rankings between faculty scores and E4D Compare scores was measured with Spearman’s correlation coefficient (SCC) at five different tolerance levels (0.1–0.5 mm).	SCC values for practical exams varied between 0.20 and 0.56. None of the upper 95% confidence limits reached the for strong correlation. SCC values indicated only weak to moderate agreement in ranks between practical exam scores and scores obtained with E4D Compare. When ranked from lowest to highest, the results from the conventional grading by the faculty did not correlate within an acceptable range to E4D Compare software data.
Mays et al. 2016 [42]	CCS	P	50	Validated E4D software (Planmeca) to assess occlusal convergence (TOC) of 50 molar crown preparations from students and compared to traditional faculty assessment.	Digital software could distinguish differences in TOC, which were grouped as minimum taper (mean 11°), moderate (mean 23°), or excessive (mean 47°). Digital TOC evaluation was more objective compared to faculty visual scoring.
Gratton et al. 2016 [45]	RCT	P	80	Compared effect of access to digital systems in addition to conventional preparation instructions; CEREC prepCheck (*n* = 20), E4D Compare (n=20), and control without access to digital system (*n* = 40); incisor and molar crown preparations were assessed by the students, by 3 faculties and by E4D Compare at 0.30 mm tolerance.	All groups had similar preparation scores. Visual and digital assessment scores showed modest correlation.
Gratton et al. 2017 [46]	RCT	P	79	Compared digital systems Compare (*n* = 42) and prepCheck (*n* = 37) as additional evaluation tool assessing their crown preparations (maxillary central incisor and mandibular molar); all preparations were graded by faculty Compare and prepCheck; feed-back with post-course questionnaire.	Both groups had similar technical scores; both systems had modest correlation with faculty scores and strong correlation with each other. 55.3% of students felt unfavorable about learning digital evaluation protocols, while 62.3% felt favorable about the integration of the tools into the curriculum.
Park et al. 2017 [44]	OT	P	36	Evaluated prepCheck for self-assessment, students performed ceramo-metal crown preparation (maxillary molar during formative exercise, mandibular molar during summative exam); five learning tools were used for assessments: reduction, margin width, surface finish, taper, undercut; tools were rated for usefulness, user-friendliness, and frequency of use (scale from 1 = lowest to 5 = highest). Faculty members graded tooth preparations as pass (P), marginal-pass (MP), or fail (F).	Tools assessing undercut and taper received highest scores for usefulness, user-friendliness, and frequency of use. Students’ performance was 38.8% P, 30.6% MP and 30.6% F. Failing students had the highest score (4.4) on usefulness.
Kateeb et al. 2017 [38]	OT	P	96	Compared digital assessment software of students’ crown preparation with traditional visual inspection; four examiners; sample of 20 preparations were reassessed for intra-rater reliability.	Intra-rater reliability (ICC) was 0.73–0.78 and 0.99 for the digital grading system; inter-rater reliability among the four examiners was good (0.76); agreement between examiners and digital ratings were low to moderate; digital grading was more consistent.
Sly et al. 2017 [50]	OT	P	98	Compared E4D software (Planmeca) to assess students intracoronal Class I preparation with traditional visual inspection; four examiners.	Similar results for grading of isthmus width and remaining marginal ridge, while pulpal floor depth was assessed more precisely with visual inspection; results indicate that software has limitations for intracoronal cavity assessment but offers a self-assessment tool to improve psychomotor skills with independent and immediate feedback.
Kunkel et al. 2018 [40]	OT	P	69	Compared prepCheck with visual faculty assessment of taper in students’ crown preparation of typodont teeth, 10 experienced course instructors.	Instructor gradings were overrated compared to digital prepCheck grades, prepCheck facilitates evaluation instantly and exactly by students and examiners.
Kozarovska & Larsson 2018 [39]	RCT	P	57	Evaluated a digital preparation validation tool (PVT) for students’ self-assessment of crown preparation (tooth 11 and 21); group A (“prep-and-scan” self-assessed and scanned three preparations; group B (“best-of-three”) self-assessed the three attempts, chose the best for scanning; questionnaire about students’ and teachers’ experiences with PVT.	Group A showed an increase in agreement of self-assessment and feedback from PVT, while group B showed low level agreement with PVT. Bucco-incisal reduction, reduction of the tuberculum surface and presence of undercuts were difficult to correctly identify by the students. Questionnaire feedback revealed need for PVT to develop skills, to ease assessment, while critical aspects were PVT’s time efficiency and the need for verbal feedback. Teachers observed the PVT as a motivation during skills laboratory training, while verbal feedback were still deemed necessary.
Wolgin et al. 2018 [53]	RCT	P	47	Investigated digital self-assessment concept (prepCheck software) for students in the phantom course preparing a three surface (MOD) class II amalgam cavity; intervention group (IG): compared a 3D image of their preparation against master preparation with PrepCheck; control group (CG): received verbal feedback from supervisor based on pre-defined criteria.	Test and control groups performed similar and self-assessment learning tool was deemed equivalent to conventional supervision.
Lee et al. 2018 [51]	OT	P	69	Compared students’ self-assessment (conventional and digital with Cerec software) with assessment (conventional and digital) by faculty members for class II amalgam preparations (C2AP) and Class III composite preparations (C3CP).	Students overestimated their performance (positive S-F gap) in both the C2AP and C3CP preparation exercises in conventional (11% and 5%) and digital assessments (8% and 2%); in conventional assessments, preclinical performance was negatively correlated with student-faculty gap (r = −0.47, *p* < 0.001); particularly students in the bottom quartile sig. improved their self-assessment accuracy using digital self-assessments over conventional assessments.
Nagy et al. 2018 [52]	RCT	P	36	Investigated the effect of a digital feedback (test group) for mesio-occlusal onlay preparation by a 3D visualization of the cavity (Dental Teacher software, KaVo), while verbal feedback from supervisor was given to control group. Following feedbacks, 2nd corrective preparations were conducted and improvements measured. Parameters: occlusal cavity depth (OD), approximal depth (AD), extent of cusp reduction on the mesiobuccal cusp (CR), width of shoulder preparation around the mesiobuccal cusp (SW), cavity width at two different points in the occlusal box (OW).	Test group improved in all parameter and showed significantly smaller deviations of mean OD, AD and mean SW; in control group, parameter deviations were similar during 1st and 2nd preparation.
Liu et al. 2018 [41]	RCT	P	66	Evaluated the effectiveness of preclinical training on ceramic crown preparation using digital training system compared with traditional training method; test group: trained with digital method with Online Peer-Review System (OPRS) and Real-time Dental Training and Evaluation System (RDTES); control group: traditional method with instructor demonstration and evaluation; central incisor crown preparation.	Five of 15 assessed items were significantly better in test group; 96.97% of test students agreed or strongly agreed that using digital training system could better improve the practical ability than traditional method.
Greany et al. 2019 [36]	OT	P	67	Compared conventional visual faculty inspection of wax-ups to digital assessment; six examiners evaluated 67 students’ wax-ups of maxillary first molar, reevaluation after 1 week; scan with IOS, STL files imported to free available open source data cloud comparison utility (Cloud Compare.org), digital evaluation by two examiners.	Visual inspection had low inter-examiner precision (ICC 0.332) and accuracy; intra-examiner precision for reevaluation was low; inter-examiner precision of digital exam was high (ICC 0.866) with high accuracy.
Miyazone et al. 2019 [43]	OT	P	100	Compared prepCheck with visual faculty assessment of students’ crown preparation of typodont teeth (mandibular first molar as crown abutment, maxillary 2nd premolar and 2nd molar as FDP abutments), assess inter- and intra-grader agreement of five experienced examiners conducting visual and digital exam; scoring repeated three times; parameters for crown abutments: axial tissue removal, margin width, undercut, occlusal reduction, cusp tips, occlusal anatomy; for FDP abutments: path of insertion.	Intra-grader agreement was better with prepCheck than visual assessment for all parameters except cusp tip and occlusal anatomy; inter-grader agreement for path of insertion was questionable with visual, but good with digital assessment. Inter-grader disagreement was greater in visual than digital assessment. Overestimation of tooth reduction in visual grading was eliminated by digital analysis.

RCT = Randomized Controlled Trial; CT = Controlled Trial; CS = Cohort Study; CCS = Case-Control-Study; OT = Observational Study; ICC = Inter-Class Correlation; STL = Standard Tessellation Language.

**Table 4:** Group 4: 3D printing and prototyping (*n* = 2).

Study (Year)	Study Design	Theory/Practice	Participants	Materials and Methods	Results
Soares et al. 2013 [77]	OT	T	40	Cavity preparation was taught with conventional teaching materials with 2D schematic illustration and photographs. New didactic material with virtual 3D (videos of the preparations) and magnified nylon prototyped models was introduced. Evaluation by questionnaire.	Improvement of teaching quality when combining 3D virtual technology with real models.
Kröger et al. 2016 [78]	OT	P	22	3D printed simulation models based on real patient situations were used for hands-on practice. Models simulated realistic tooth positions and wide variability of dental cases and procedures. Students removed a crown from tooth 16, detected and removed caries, did a build-up filling and crown preparation within 3 h. Students’ feedback on a VAS questionnaire.	Students evaluated models based on real patient situations as good training possibilities. The lack of gingiva was disturbing.

RCT = Randomized Controlled Trial; CT = Controlled Trial; CS = Cohort Study; CCS = Case-Control-Study; OT = Observational Study.

**Table 5:** Group 5: Digital Radiology (*n* = 5).

Study (Year)	Study Design	Theory/Practice	Participants	Materials and Methods	Results
Mileman et al. 2003 [79]	RCT	P	67	Investigated computer-assisted learning (CAL) calibration program to improves dental students’ accuracy in dentin caries detection from bitewing radiographs; experimental (*n* = 33) group: used CAL with feedback for self-calibration control (*n* = 34) group.	CAL improved students’ diagnostic performance; true positive ratio (sensitivity) for caries detection was significantly higher in test 76.3% than control with 66.9%, while false positive ratio (specificity) was similar (28.1 and 28.7%); diagnostic odds ratio was sig. higher in test (12.4) than in control (8.8).
Wenzel et al. 2004 [83]	RCT	P	31	Compared 2 digital systems (RVG-ui CCD sensor, Digora PSP plate system) for radiographic examination; after education in digital radiography one student group started with CCD, one with PSP and both completed endodontic treatment of single-rooted extracted tooth; groups switched radiography system and treated a 2nd tooth. True tooth length (TTL) and root filling length (RFL) were measured with the software and compared to manual measurement; feed-back questionnaire after each treatment.	Using CCD sensor required less time than PSP; positioning the tooth was easier with PSP plate; positive attitudes towards digital radiography; lengths measured on the digital images from both digital systems were slightly larger than true tooth lengths with no difference in ratio TTL/RFL between systems.
Minston et al. 2013 [80]	CT	P	20	Investigated students’ diagnostic performance on approximal caries detection with analog and digital radiographs from 46 extracted human premolars and molars, compared diagnostic accuracy; teeth were sectioned and histopathologically analyzed (gold standard)	Students ability for caries detection was poor, no difference between analog and digital radiographs.
Busanello et al. 2015 [81]	CCS	P	62	Evaluated digital learning object to improve skills in diagnosing radiographic dental changes (Visual Basic Application software); test group used the digital tool, control group: conventional imaging diagnosis course; diagnosis test after 3 weeks.	Test group performed significantly better, females were better than males.
Kratz et al. 2018 [82]	CT	P	169	Evaluated students’ ability to identify positional errors (tongue position, head rotation, chin position) in panoramic radiographs of edentulous patients, students in 2nd year (*n* = 84) and 3rd–4th year (*n* = 85)	2nd year students identified significantly more positional errors than 3rd and 4th students. Students were more experienced at identifying radiographic findings compared to positional errors.

RCT = Randomized Controlled Trial; CT = Controlled Trial; CS = Cohort Study; CCS = Case-Control-Study; OT = Observational Study; CCD = Charged Couple Device; PSP = Photostimulable Phosphor.

**Table 6:** Surveys related to digital education (*n* = 10).

Study (Year)	Study Design	Theory/Practice	Participants	Materials and Methods	Results
Scarfe et al. 1996 [88]	OT	T	277	Investigated the effects of instructions in intraoral digital radiology on dental students’ knowledge, attitudes and beliefs; 174 from a university with formal instruction on digital dental radiography, and 103 from a university without instructions.	Students with instructions knew significantly more than students without; 93% wanted digital radiology to be included in the dental curriculum.
McCann et al. 2010 [85]	OT	T	366	Surveyed student’s (dental and dental hygiene) preferences for e-teaching and learning, using an online questionnaire in 2008 related to computer experience, use and effectiveness of e-resources, preferences for various environments, need for standardization, and preferred modes of communication.	64% preferred printed text over digital and 74% wanted e-materials to supplement but not replace lectures; 71% preferred buying traditional textbooks, 11% preferred electronic versions; among e-resources virtual microscopy (69%), digital skull atlas (68%), and digital tooth atlas (64%) were reported as most effective; e-materials would enhance learning, in particular e-lectures (59%), clinical videos (54%), and podcasts (45%). E-resources should not replace interactions with faculty; students wanted lectures and clinical procedures recorded.
Jathanna et al. 2014 [84]	OT	T	186	Surveyed the perception of Indian dental students toward usefulness of digital technologies in improving dental practice, willingness to use digital and electronic technologies, perceived obstacles to use digital and electronic technologies in dental care setups, and their attitudes toward internet privacy issues.	Students indicated that digital technology increases patient satisfaction and practice efficiency, improves record quality, doctor-doctor communication, case diagnosis and treatment planning; obstacles to the wide adoption of these technologies were cost and dentists’ lack of knowledge and comfort with technology.
Chatham et al. 2014 [90]	OT	T	11	Surveyed the penetration of digital technologies in UK dental schools (11/16 responded).	45% did not teach digital technologies (36% because it was not part of the curriculum, or in 95% due to the lack of technical expertise or support); half of those teaching digital technologies did so with lectures or demonstrations, the other half allowed practical involvement.
Brownstein et al. 2015 [91]	OT	T	33	Surveyed the penetration of emerging dental technologies into the curricula at US dental schools (62 eligible schools were contacted); academic Deans answered 19 questions related to 12 dental topics); 19 schools had <100 students/class; 14 had >100 students.	Highest penetration was in preclinical didactic courses (62%) and lowest was in preclinical laboratory (36%); most common specific technologies were digital radiography (85%) and rotary endodontics (81%), least common were CAD/CAM denture fabrication (20%) and hard tissue lasers (24%); the bigger the class sizes (>100 students) and the older the school, the lower the incorporation of newer technologies.
Bhardwaj et al. 2015 [92]	OT	T	54	Surveyed faculties’ opinion (15 dental, 42 medical faculty members in Melaka, Malaysia) toward the existing e-learning activities, and to analyze the extent of adopting and integration of e-learning into their traditional teaching methods; questionnaire with socio-demographic profile, skills and aptitude on the use of computer, knowledge and use of existing e-learning technology (e.g., MOODLE), experiences and attitudes towards e-learning, faculty opinion on novel e-learning techniques, and initiatives to be adopted for optimization of existing e-learning facilities.	65.4% of faculty was positive towards e-learning; formal training required to support e-learning that enables smooth transition of the faculty from traditional teaching into blended approach; traditional instructor centered teaching is shifting to learner centered model facilitating students to control their own learning. Popular e-learning education tools: Virtual Learning Environment systems such as WebCT™.
Ren et al. 2017 [86]	OT	T	389	Questionnaire assessed students’ attitudes towards digital simulation technologies and teaching methods, how students compare digital technologies with traditional training methods; four categories: digital microscope, virtual pathology slides, digital radiology, virtual simulation training.	Most students accepted digital technologies as stimulating tool for self-learning; digital X-ray images were used to study oral radiology and preferred to conventional X-rays. Dental simulation training was most preferred technology (54.6%), 16.7% preferred digital microscopy, 15.0% virtual pathology slides, 13.7% digital x-ray images. 76% used the virtual simulation training machine to study oral clinical skills; 61% felt that the simulator would be a useful addition to current pre-clinical training; 66% felt that the simulator provided a realistic virtual environment.
Roberts et al. 2019 [87]	OT	T	282 (in 2015) 129 (in 2017)	Surveyed the use of student-managed online technologies in collaborative e-learning; comparison of web-based applications and other study methods (survey in 2015 focused on Google Doc/survey in 2017 focused on all e-learning technologies).	Significant decrease in Google Docs overall usage in 2017 (95%) compared to 2015 (99%), but significantly increased frequency of use in all courses from 36% (2015) to 71.6% (2017). The use of textbooks dropped significantly from 25% (2015) to 15% (2017). Only 4% reported that textbooks were worth the cost. 52% would not use textbooks to study even when placed at disposal. In 2017 52% spent study time with social media (Twitter or Facebook), 66% “sometimes” questioned the validity of information posted by others in collaborative documents. To collaboratively study with peers, Google Docs and personal contacts were the top choices in 2017.
Prager & Liss 2019 [2]	OT	T	54	Surveyed the extent of teaching digital modalities and use for patient care in dental schools (54 out of 76 dental schools in U.S. and Canada responded) in February 2019.	93% used CAD/CAM digital scanning, IOS was performed exclusively in 55%, extraoral model scan was used as sole technique in 8%, intra- and extraoral scanning in 37% of the schools. IOS was applied for crowns (100%), inlays/onlays (77%), implant crowns (52%), fixed partial denture (34%), complete denture (2%), but none of the schools indicated to use IOS always for crowns. 59% had a digital workflow established to deliver same-day restorations. 34% had at least 10% of faculty proficient in IOS, 66% had 10% or less.
Turkyilmaz et al. 2019 [89]	OT	T	255	Surveyed students’ perception of e-learning impact on dental education, response rate of 22.6% (255 out of 1130 electronically distributed 14-question surveys to 2nd–4th year students).	48.6% preferred traditional lecture mixed with online learning, 18.4% online classes only, 18.0% traditional lecture style only; greatest impact on learning had YouTube, Bone Box, and Google. 60% spent between 1 and >4 h per day on electronic resources for academic performance. E-learning had a significant perceived effect on didactic and clinical understanding. Students observed that faculties estimated <50 years of age were more likely to incorporate e-learning into courses and more likely to use social media for communication.

RCT = Randomized Controlled Trial; CT = Controlled Trial; CS = Cohort Study; CCS = Case-Control-Study; OT = Observational Study.

## Supplementary Materials

The following are available online at https://www.mdpi.com/1660-4601/17/9/3269/s1, Annex S1 and Annex S2.
