# Supplementary material for: Digital Undergraduate Education in Dentistry: A Systematic Review
Source: Int J Environ Res Public Health. 2020 May 7;17(9):3269. doi: 10.3390/ijerph17093269 (PMC7246576; doi:10.3390/ijerph17093269)
Supplement: Supplementary file 1 [file ijerph-17-03269-s001.pdf]

Annex I: Included full-texts based on the systematic search

- Alves, L.S.; de Oliveira, R.S.; Nora, A.D.; Cuozzo Lemos, L.F.; Rodrigues, J.A.; Zenkner, J.E.A. Dental Students' Performance in Detecting In Vitro Occlusal Carious Lesions Using ICDAS with E-Learning and Digital Learning Strategies. *J. Dent. Educ.* **2018**, *82*, 1077–1083, doi:10.21815/JDE.018.100.
- Bains, M.; Reynolds, P.A.; McDonald, F.; Sherriff, M. Effectiveness and acceptability of face-toface,blended and e-learning: a randomised trial of orthodontic undergraduates. *Eur. J. Dent. Educ.* **2011**, *15*, 110–117, doi:10.1111/j.1600-0579.2010.00651.x.
- Ben-Gal, G.; Weiss, E.I.; Gafni, N.; Ziv, A. Testing manual dexterity using a virtual reality simulator: reliability and validity. *Eur. J. Dent. Educ.* **2013**, *17*, 138–142, doi:10.1111/eje.12023.
- Bhardwaj, A.; Nagandla, K.; Swe, K.M.; Abas, A.B. Academic Staff Perspectives Towards Adoption of E-learning at Melaka Manipal Medical College: Has E-learning Redefined our Teaching Model? *Kathmandu Univ. Med. J. (KUMJ)* **2015**, *13*, 12–18, doi:10.3126/kumj.v13i1.13746.
- Botelho, J.; Machado, V.; Proenca, L.; Rua, J.; Delgado, A.; Joao Mendes, J. Cloud-based collaboration and productivity tools to enhance self-perception and self-evaluation in senior dental students: A pilot study. *Eur. J. Dent. Educ.* **2019**, *23*, e53–e58, doi:10.1111/eje.12400.
- Boynton, J.R.; Johnson, L.A.; Nainar, S.M.; Hu, J.C. Portable digital video instruction in predoctoral education of child behavior management. *J. Dent. Educ.* **2007**, *71*, 545–549.
- Brownstein, S.A.; Murad, A.; Hunt, R.J. Implementation of new technologies in U.S. dental school curricula. *J. Dent. Educ.* **2015**, *79*, 259–264.
- Busanello, F.H.; da Silveira, P.F.; Liedke, G.S.; Arus, N.A.; Vizzotto, M.B.; Silveira, H.E.; Silveira, H.L. Evaluation of a digital learning object (DLO) to support the learning process in radiographic dental diagnosis. *Eur. J. Dent. Educ.* **2015**, *19*, 222–228, doi:10.1111/eje.12125.
- Callan, R.S.; Haywood, V.B.; Cooper, J.R.; Furness, A.R.; Looney, S.W. The Validity of Using E4D Compare's "% Comparison" to Assess Crown Preparations in Preclinical Dental Education. *J. Dent. Educ.* **2015**, *79*, 1445–1451.
- Chatham, C.; Spencer, M.H.; Wood, D.J.; Johnson, A. The introduction of digital dental technology into BDS curricula. *Br. Dent. J.* **2014**, *217*, 639-642, doi:10.1038/sj.bdj.2014.1049.
- Curnier, F. Teaching dentistry by means of virtual reality--the Geneva project. *Int. J. Comput. Dent.* **2010**, *13*, 251–263.
- de Boer, I.R.; Lagerweij, M.D.; Wesselink, P.R.; Vervoorn, J.M. The Effect of Variations in Force Feedback in a Virtual Reality Environment on the Performance and Satisfaction of Dental Students. *Simul. Healthc.* **2019**, *14*, 169–174, doi:10.1097/SIH.0000000000000370.
- Douglas, R.D.; Hopp, C.D.; Augustin, M.A. Dental students' preferences and performance in crown design: conventional wax-added versus CAD. *J. Dent. Educ.* **2014**, *78*, 1663–1672.
- Esser, C.; Kerschbaum, T.; Winkelmann, V.; Krage, T.; Faber, F.J. A comparison of the visual and technical assessment of preparations made by dental students. *Eur. J. Dent. Educ.* **2006**, *10*, 157–161, doi:10.1111/j.1600-0579.2006.00408.x.
- Gal, G.B.; Weiss, E.I.; Gafni, N.; Ziv, A. Preliminary assessment of faculty and student perception of a haptic virtual reality simulator for training dental manual dexterity. *J. Dent. Educ.* **2011**, *75*, 496–504.
- Garrett, P.H.; Faraone, K.L.; Patzelt, S.B.; Keaser, M.L. Comparison of Dental Students' Self-Directed, Faculty, and Software-Based Assessments of Dental Anatomy Wax-Ups: A Retrospective Study. *J. Dent. Educ.* **2015**, *79*, 1437–1444.
- Gonzalez, S.M.; Gadbury-Amyot, C.C. Using Twitter for Teaching and Learning in an Oral and Maxillofacial Radiology Course. *J. Dent. Educ.* **2016**, *80*, 149–155.
- Gottlieb, R.; Lanning, S.K.; Gunsolley, J.C.; Buchanan, J.A. Faculty impressions of dental students' performance with and without virtual reality simulation. *J. Dent. Educ.* **2011**, *75*, 1443–1451.
- Gratton, D.G.; Kwon, S.R.; Blanchette, D.; Aquilino, S.A. Impact of Digital Tooth Preparation Evaluation Technology on Preclinical Dental Students' Technical and Self-Evaluation Skills. *J. Dent. Educ.* **2016**, *80*, 91–99.

- Gratton, D.G.; Kwon, S.R.; Blanchette, D.R.; Aquilino, S.A. Performance of two different digital evaluation systems used for assessing pre-clinical dental students' prosthodontic technical skills. *Eur. J. Dent. Educ.* **2017**, *21*, 252–260, doi:10.1111/eje.12231.
- Greany, T.J.; Yassin, A.; Lewis, K.C. Developing an All-Digital Workflow for Dental Skills Assessment: Part I, Visual Inspection Exhibits Low Precision and Accuracy. *J. Dent. Educ.* **2019**, *83*, 1304–1313, doi:10.21815/JDE.019.132.
- Hamil, L.M.; Mennito, A.S.; Renne, W.G.; Vuthiganon, J. Dental students' opinions of preparation assessment with E4D compare software versus traditional methods. *J. Dent. Educ.* **2014**, *78*, 1424–1431.
- Jackson, T.H.; Zhong, J.; Phillips, C.; Koroluk, L.D. Self-Directed Digital Learning: When Do Dental Students Study? *J. Dent. Educ.* **2018**, *82*, 373–378, doi:10.21815/jde.018.040.
- Jasinevicius, T.R.; Landers, M.; Nelson, S.; Urbankova, A. An evaluation of two dental simulation systems: virtual reality versus contemporary non-computer-assisted. *J. Dent. Educ.* **2004**, *68*, 1151–1162.
- Jathanna, V.R.; Jathanna, R.V.; Jathanna, R. The awareness and attitudes of students of one indian dental school toward information technology and its use to improve patient care. *Educ. Health (Abingdon)* **2014**, *27*, 293–296, doi:10.4103/1357-6283.152194.
- Kateeb, E.T.; Kamal, M.S.; Kadamani, A.M.; Abu Hantash, R.O.; Abu Arqoub, M.M. Utilising an innovative digital software to grade pre-clinical crown preparation exercise. *Eur. J. Dent. Educ.* **2017**, *21*, 220–227, doi:10.1111/eje.12204.
- Kattadiyil, M.T.; Jekki, R.; Goodacre, C.J.; Baba, N.Z. Comparison of treatment outcomes in digital and conventional complete removable dental prosthesis fabrications in a predoctoral setting. *J. Prosthet. Dent.* **2015**, *114*, 818–825, doi:10.1016/j.prosdent.2015.08.001.
- Kikuchi, H.; Ikeda, M.; Araki, K. Evaluation of a virtual reality simulation system for porcelain fused to metal crown preparation at Tokyo Medical and Dental University. *J. Dent. Educ.* **2013**, *77*, 782–792.
- Kingsley, K.V.; Kingsley, K. A case study for teaching information literacy skills. *BMC Med. Educ.* **2009**, *9*, 7, doi:10.1186/1472-6920-9-7.
- Komolpis, R.; Johnson, R.A. Web-based orthodontic instruction and assessment. *J. Dent. Educ.* **2002**, *66*, 650-658.
- Kozarovska, A.; Larsson, C. Implementation of a digital preparation validation tool in dental skills laboratory training. *Eur. J. Dent. Educ.* **2018**, *22*, 115–121, doi:10.1111/eje.12272.
- Kratz, R.J.; Nguyen, C.T.; Walton, J.N.; MacDonald, D. Dental Students' Interpretations of Digital Panoramic Radiographs on Completely Edentate Patients. *J. Dent. Educ.* **2018**, *82*, 313–321, doi:10.21815/JDE.018.033.
- Kroger, E.; Dekiff, M.; Dirksen, D. 3D printed simulation models based on real patient situations for hands-on practice. *Eur J. Dent. Educ.* **2017**, *21*, e119–e125, doi:10.1111/eje.12229.
- Kunkel, T.C.; Engelmeier, R.L.; Shah, N.H. A comparison of crown preparation grading via PrepCheck versus grading by dental school instructors. *Int. J. Comput. Dent.* **2018**, *21*, 305–311.
- Kwon, S.R.; Restrepo-Kennedy, N.; Dawson, D.V.; Hernandez, M.; Denehy, G.; Blanchette, D.; Gratton, D.G.; Aquilino, S.A.; Armstrong, S.R. Dental anatomy grading: comparison between conventional visual and a novel digital assessment technique. *J. Dent. Educ.* **2014**, *78*, 1655–1662.
- LeBlanc, V.R.; Urbankova, A.; Hadavi, F.; Lichtenthal, R.M. A preliminary study in using virtual reality to train dental students. *J. Dent. Educ.* **2004**, *68*, 378–383.
- Lee, C.; Kobayashi, H.; Lee, S.R.; Ohyama, H. The Role of Digital 3D Scanned Models in Dental Students' Self-Assessments in Preclinical Operative Dentistry. *J. Dent. Educ.* **2018**, *82*, 399–405, doi:10.21815/jde.018.046.
- Lee, S.J.; Gallucci, G.O. Digital vs. conventional implant impressions: efficiency outcomes. *Clin. Oral Implants Res.* **2013**, *24*, 111–115, doi:10.1111/j.1600-0501.2012.02430.x.

- Liu, L.; Li, J.; Yuan, S.; Wang, T.; Chu, F.; Lu, X.; Hu, J.; Wang, C.; Yan, B.; Wang, L. Evaluating the effectiveness of a preclinical practice of tooth preparation using digital training system: A randomised controlled trial. *Eur. J. Dent. Educ.* **2018**, *22*, e679–e686, doi:10.1111/eje.12378.
- Luz, P.B.; Stringhini, C.H.; Otto, B.R.; Port, A.L.; Zaleski, V.; Oliveira, R.S.; Pereira, J.T.; Lussi, A.; Rodrigues, J.A. Performance of undergraduate dental students on ICDAS clinical caries detection after different learning strategies. *Eur. J. Dent. Educ.* **2015**, *19*, 235–241, doi:10.1111/eje.12131.
- Mahrous, A.; Schneider, G.B.; Holloway, J.A.; Dawson, D.V. Enhancing Student Learning in Removable Partial Denture Design by Using Virtual Three-Dimensional Models Versus Traditional Two-Dimensional Drawings: A Comparative Study. *J. Prosthodont.* **2019**, *28*, 927–933, doi:10.1111/jopr.13099.
- Marti, A.M.; Harris, B.T.; Metz, M.J.; Morton, D.; Scarfe, W.C.; Metz, C.J.; Lin, W.S. Comparison of digital scanning and polyvinyl siloxane impression techniques by dental students: instructional efficiency and attitudes towards technology. *Eur J. Dent. Educ.* **2017**, *21*, 200–205, doi:10.1111/eje.12201.
- Mays, K.A.; Crisp, H.A.; Vos, P. Utilizing CAD/CAM to Measure Total Occlusal Convergence of Preclinical Dental Students' Crown Preparations. *J. Dent. Educ.* **2016**, *80*, 100–107.
- Mays, K.A.; Levine, E. Dental students' self-assessment of operative preparations using CAD/CAM: a preliminary analysis. *J. Dent. Educ.* **2014**, *78*, 1673–1680.
- McCann, A.L.; Schneiderman, E.D.; Hinton, R.J. E-teaching and learning preferences of dental and dental hygiene students. *J. Dent. Educ.* **2010**, *74*, 65–78.
- Mileman, P.A.; van den Hout, W.B.; Sanderink, G.C. Randomized controlled trial of a computer-assisted learning program to improve caries detection from bitewing radiographs. *Dentomaxillofac. Radiol.* **2003**, *32*, 116–123, doi:10.1259/dmfr/58225203.
- Minston, W.; Li, G.; Wennberg, R.; Nasstrom, K.; Shi, X.Q. Comparison of diagnostic performance on approximal caries detection among Swedish and Chinese senior dental students using analogue and digital radiographs. *Swed. Dent. J.* **2013**, *37*, 79–85.
- Mitov, G.; Dillschneider, T.; Abed, M.R.; Hohenberg, G.; Pospiech, P. Introducing and evaluating MorphoDent, a Web-based learning program in dental morphology. *J. Dent. Educ.* **2010**, *74*, 1133–1139.
- Miyazono, S.; Shinozaki, Y.; Sato, H.; Isshi, K.; Yamashita, J. Use of Digital Technology to Improve Objective and Reliable Assessment in Dental Student Simulation Laboratories. *J. Dent. Educ.* **2019**, *83*, 1224–1232, doi:10.21815/JDE.019.114.
- Murbay, S.; Neelakantan, P.; Chang, J.W.W.; Yeung, S. "Evaluation of the introduction of a dental virtual simulator on the performance of undergraduate dental students in the pre-clinical operative dentistry course". *Eur. J. Dent. Educ.* **2020**, doi:10.1111/eje.12453.
- Murrell, M.; Marchini, L.; Blanchette, D.; Ashida, S. Intraoral Camera Use in a Dental School Clinic: Evaluations by Faculty, Students, and Patients. *J. Dent. Educ.* **2019**, *83*, 1339–1344, doi:10.21815/JDE.019.140.
- Nagy, Z.A.; Simon, B.; Toth, Z.; Vag, J. Evaluating the efficiency of the Dental Teacher system as a digital preclinical teaching tool. *Eur J. Dent. Educ.* **2018**, *22*, e619–e623, doi:10.1111/eje.12365.
- Park, C.F.; Sheinbaum, J.M.; Tamada, Y.; Chandiramani, R.; Lian, L.; Lee, C.; Da Silva, J.; Ishikawa-Nagai, S. Dental Students' Perceptions of Digital Assessment Software for Preclinical Tooth Preparation Exercises. *J. Dent. Educ.* **2017**, *81*, 597–603, doi:10.21815/jde.016.015.
- Pohlenz, P.; Grobe, A.; Petersik, A.; von Sternberg, N.; Pflesser, B.; Pommert, A.; Hohne, K.H.; Tiede, U.; Springer, I.; Heiland, M. Virtual dental surgery as a new educational tool in dental school. *J. Craniomaxillofac. Surg.* **2010**, *38*, 560–564, doi:10.1016/j.jcms.2010.02.011.
- Prager, M.C.; Liss, H. Assessment of Digital Workflow in Predoctoral Education and Patient Care in North American Dental Schools. *J. Dent. Educ.* **2019**, doi:10.21815/JDE.019.177.

- Pyorala, E.; Maenpaa, S.; Heinonen, L.; Folger, D.; Masalin, T.; Hervonen, H. The art of note taking with mobile devices in medical education. *BMC Med. Educ.* **2019**, *19*, 96, doi:10.1186/s12909-019-1529-7.
- Qi, S.; Yan, Y.; Li, R.; Hu, J. The impact of active versus passive use of 3D technology: a study of dental students at Wuhan University, China. *J. Dent. Educ.* **2013**, *77*, 1536–1542.
- Quinn, F.; Keogh, P.; McDonald, A.; Hussey, D. A study comparing the effectiveness of conventional training and virtual reality simulation in the skills acquisition of junior dental students. *Eur. J. Dent. Educ.* **2003**, *7*, 164–169, doi:10.1034/j.1600-0579.2003.00309.x.
- Rees, J.S.; Jenkins, S.M.; James, T.; Dummer, P.M.; Bryant, S.; Hayes, S.J.; Oliver, S.; Stone, D.; Fenton, C. An initial evaluation of virtual reality simulation in teaching pre-clinical operative dentistry in a UK setting. *Eur. J. Prosthodont. Restor. Dent.* **2007**, *15*, 89–92.
- Reissmann, D.R.; Sierwald, I.; Berger, F.; Heydecke, G. A model of blended learning in a preclinical course in prosthetic dentistry. *J. Dent. Educ.* **2015**, *79*, 157–165.
- Ren, Q.; Wang, Y.; Zheng, Q.; Ye, L.; Zhou, X.D.; Zhang, L.L. Survey of student attitudes towards digital simulation technologies at a dental school in China. *Eur. J. Dent. Educ.* **2017**, *21*, 180–186, doi:10.1111/eje.12198.
- Reynolds, P.A.; Harper, J.; Dunne, S.; Cox, M.; Myint, Y.K. Portable digital assistants (PDAs) in dentistry: part II--pilot study of PDA use in the dental clinic. *Br. Dent. J.* **2007**, *202*, 477–483, doi:10.1038/bdj.2007.296.
- Roberts, B.S.; Roberts, E.P.; Reynolds, S.; Stein, A.F. Dental Students' Use of Student-Managed Google Docs and Other Technologies in Collaborative Learning. *J. Dent. Educ.* **2019**, *83*, 437–444, doi:10.21815/jde.019.053.
- Scarfe, W.C.; Potter, B.J.; Farman, A.G. Effects of instruction on the knowledge, attitudes and beliefs of dental students towards digital radiography. *Dentomaxillofac Radiol.* **1996**, *25*, 103–108, doi:10.1259/dmfr.25.2.9446981.
- Schittek Janda, M.; Mattheos, N.; Nattestad, A.; Wagner, A.; Nebel, D.; Farbom, C.; Le, D.H.; Attstrom, R. Simulation of patient encounters using a virtual patient in periodontology instruction of dental students: design, usability, and learning effect in history-taking skills. *Eur. J. Dent. Educ.* **2004**, *8*, 111–119, doi:10.1111/j.1600-0579.2004.00339.x.
- Schott, T.C.; Arsalan, R.; Weimer, K. Students' perspectives on the use of digital versus conventional dental impression techniques in orthodontics. *BMC Med. Educ.* **2019**, *19*, 81, doi:10.1186/s12909-019-1512-3.
- Schultze-Mosgau, S.; Zielinski, T.; Lochner, J. Web-based, virtual course units as a didactic concept for medical teaching. *Med. Teach.* **2004**, *26*, 336–342, doi:10.1080/01421590410001679028.
- Schwindling, F.S.; Deisenhofer, U.K.; Porsche, M.; Rammelsberg, P.; Kappel, S.; Stober, T. Establishing CAD/CAM in Preclinical Dental Education: Evaluation of a Hands-On Module. *J. Dent. Educ.* **2015**, *79*, 1215–1221.
- Sly, M.M.; Barros, J.A.; Streckfus, C.F.; Arriaga, D.M.; Patel, S.A. Grading Class I Preparations in Preclinical Dental Education: E4D Compare Software vs. the Traditional Standard. *J. Dent. Educ.* **2017**, *81*, 1457–1462, doi:10.21815/JDE.017.107.
- Smith, W.; Rafeek, R.; Marchan, S.; Paryag, A. The use of video-clips as a teaching aide. *Eur. J. Dent. Educ.* **2012**, *16*, 91–96, doi:10.1111/j.1600-0579.2011.00724.x.
- Soares, P.V.; de Almeida Milito, G.; Pereira, F.A.; Reis, B.R.; Soares, C.J.; de Sousa Menezes, M.; de Freitas Santos-Filho, P.C. Rapid prototyping and 3D-virtual models for operative dentistry education in Brazil. *J. Dent. Educ.* **2013**, *77*, 358–363.
- Turkyilmaz, I.; Hariri, N.H.; Jahangiri, L. Student's Perception of the Impact of E-learning on Dental Education. *J. Contemp. Dent. Pract.* **2019**, *20*, 616–621.
- Urbankova, A. Impact of computerized dental simulation training on preclinical operative dentistry examination scores. *J. Dent. Educ.* **2010**, *74*, 402–409.
- Vuchkova, J.; Maybury, T.; Farah, C.S. Digital interactive learning of oral radiographic anatomy. *Eur J. Dent. Educ.* **2012**, *16*, e79–e87, doi:10.1111/j.1600-0579.2011.00679.x.

- Wang, D.; Zhao, S.; Li, T.; Zhang, Y.; Wang, X. Preliminary evaluation of a virtual reality dental simulation system on drilling operation. *Biomed. Mater. Eng.* **2015**, *26 Suppl 1*, S747–S756, doi:10.3233/BME-151366.
- Weaver, J.M.; Lu, M.; McCloskey, K.L.; Herndon, E.S.; Tanaka, W. Digital multimedia instruction enhances teaching oral and maxillofacial suturing. *J. Calif. Dent. Assoc.* **2009**, *37*, 859–862.
- Wegner, K.; Michel, K.; Seelbach, P.H.; Wostmann, B. A questionnaire on the use of digital denture impressions in a preclinical setting. *Int. J. Comput. Dent.* **2017**, *20*, 177–192.
- Welk, A.; Maggio, M.P.; Simon, J.F.; Scarbecz, M.; Harrison, J.A.; Wicks, R.A.; Gilpatrick, R.O. Computer-assisted learning and simulation lab with 40 DentSim units. *Int. J. Comput. Dent.* **2008**, *11*, 17–40.
- Wenzel, A.; Kirkevang, L.L. Students' attitudes to digital radiography and measurement accuracy of two digital systems in connection with root canal treatment. *Eur J. Dent. Educ.* **2004**, *8*, 167–171, doi:10.1111/j.1600-0579.2004.00347.x.
- Wolgin, M.; Grabowski, S.; Elhadad, S.; Frank, W.; Kielbassa, A.M. Comparison of a prepCheck-supported self-assessment concept with conventional faculty supervision in a pre-clinical simulation environment. *Eur J. Dent. Educ.* **2018**, *22*, e522–e529, doi:10.1111/eje.12337.
- Wright, E.F.; Hendricson, W.D. Evaluation of a 3-D interactive tooth atlas by dental students in dental anatomy and endodontics courses. *J. Dent. Educ.* **2010**, *74*, 110–122.
- Zitzmann, N.U.; Kovaltschuk, I.; Lenherr, P.; Dedem, P.; Joda, T. Dental Students' Perceptions of Digital and Conventional Impression Techniques: A Randomized Controlled Trial. *J. Dent. Educ.* **2017**, *81*, 1227–1232, doi:10.21815/jde.017.081.

Annex II: Excluded full-texts based on the systematic search

- Al-Omari, W.M.; Al-Wahadni, A.M. Convergence angle, occlusal reduction, and finish line depth of full-crown preparations made by dental students. *Quintessence Int.* **2004**, *35*, 287–293.
- de Peralta, T.L.; Farrior, O.F.; Flake, N.M.; Gallagher, D.; Susin, C.; Valenza, J. The Use of Social Media by Dental Students for Communication and Learning: Two Viewpoints: Viewpoint 1: Social Media Use Can Benefit Dental Students' Communication and Learning and Viewpoint 2: Potential Problems with Social Media Outweigh Their Benefits for Dental Education. *J. Dent. Educ.* **2019**, *83*, 663–668, doi:10.21815/jde.019.072.
- Goodacre, C.J. Digital Learning Resources for Prosthodontic Education: The Perspectives of a Long-Term Dental Educator Regarding 4 Key Factors. *J. Prosthodont.* **2018**, *27*, 791–797, doi:10.1111/jopr.12987.
- Hu, W.J. [A glance at the American training mode of the clinical postgraduate students of periodontology through postdoctoral program in periodontics of University of Washington School of Dentistry]. *Zhonghua Kou Qiang Yi Xue Za Zhi* **2018**, *53*, 145–149, doi:10.3760/cma.j.issn.1002-0098.2018.03.001.
- John, B.A. Kinship--king's social harmonisation project. Pilot phase of a social network for use in higher education (HE). *Bull Group Int. Rech. Sci. Stomatol. Odontol.* **2013**, *51*, e19–e22.
- Kournetas, N.; Jaeger, B.; Axmann, D.; Groten, M.; Lachmann, S.; Weber, H.; Geis-Gerstorfer, J. Assessing the reliability of a digital preparation assistant system used in dental education. *J. Dent. Educ.* **2004**, *68*, 1228–1234.
- Lam, M.T.; Kwon, S.R.; Qian, F.; Denehy, G.E. Evaluation of an Innovative Digital Assessment Tool in Dental Anatomy. *J. Contemp. Dent. Pract* **2015**, *16*, 366–371, doi:10.5005/jp-journals-10024-1691.
- Lanning, S.K.; Best, A.M.; Temple, H.J.; Richards, P.S.; Carey, A.; McCauley, L.K. Accuracy and consistency of radiographic interpretation among clinical instructors using two viewing systems. *J. Dent. Educ.* **2006**, *70*, 149–159.

- Liu, K.N.; Xie, Y.; Gao, L.; Zhao, Y.B.; Shi, D.; Zhong, J.S.; Hu, W.J.; Ouyang, X.Y. [Comparison of two training protocols in periodontal surgery teaching for undergraduate students]. *Zhonghua Kou Qiang Yi Xue Za Zhi* **2019**, *54*, 335–338, doi:10.3760/cma.j.issn.1002-0098.2019.05.008.
- Liu, X.; Liu, M.; Yang, Y.; Fan, C.; Tan, J. Step-by-step teaching method improves the learner achievement in dental skill training. *Eur J. Dent. Educ* **2019**, *23*, 344–348, doi:10.1111/eje.12435.
- Mahrous, A.; Schneider, G.B. Enhancing Student Learning of Removable Prosthodontics Using the Latest Advancements in Virtual 3D Modeling. *J. Prosthodont* **2019**, *28*, 471–472, doi:10.1111/jopr.13044.
- Seymour, B.; Yang, H.; Getman, R.; Barrow, J.; Kalenderian, E. Patient-Centered Communication: Exploring the Dentist's Role in the Era of e-Patients and Health 2.0. *J. Dent. Educ.* **2016**, *80*, 697–704.
- Tran, J.; Dudley, J.; Richards, L. All-ceramic crown preparations: An alternative technique. *Aust. Dent. J.* **2017**, *62*, 65–70, doi:10.1111/adj.12433.
- Wolgin, M.; Wiedemann, P.; Frank, W.; Wrbas, K.T.; Kielbassa, A.M. Development and Evaluation of an Endodontic Simulation Model for Dental Students. *J. Dent. Educ.* **2015**, *79*, 1363–1372.
- Zhang, W.; Abramovitch, K.; Thames, W.; Leon, I.L.; Colosi, D.C.; Goren, A.D. Comparison of the efficacy and technical accuracy of different rectangular collimators for intraoral radiography. *Oral Surg. Oral Med. Oral Pathol. Oral Radiol. Endod.* **2009**, *108*, e22–e28, doi:10.1016/j.tripleo.2009.03.011.
